# *Aeromonas* Surface Glucan Attached through the O-Antigen Ligase Represents a New Way to Obtain UDP-Glucose

**DOI:** 10.1371/journal.pone.0035707

**Published:** 2012-05-01

**Authors:** Susana Merino, Lamiaa Bouamama, Yuriy A. Knirel, Sofya N. Senchenkova, Miguel Regué, Juan M. Tomás

**Affiliations:** 1 Departamento de Microbiología, Facultad de Biología, Universidad de Barcelona, Diagonal, Barcelona, Spain; 2 N. D. Zelinsky Institute of Organic Chemistry, Russian Academy of Sciences, Moscow, Russia; 3 Departamento de Microbiología y Parasitología Sanitarias, Facultad de Farmacia, Universidad de Barcelona, Barcelona, Spain; Monash University, Australia

## Abstract

We previously reported that *A. hydrophila* GalU mutants were still able to produce UDP-glucose introduced as a glucose residue in their lipopolysaccharide core. In this study, we found the unique origin of this UDP-glucose from a branched α-glucan surface polysaccharide. This glucan, surface attached through the O-antigen ligase (WaaL), is common to the mesophilic *Aeromonas* strains tested. The *Aeromonas* glucan is produced by the action of the glycogen synthase (GlgA) and the UDP-Glc pyrophosphorylase (GlgC), the latter wrongly indicated as an ADP-Glc pyrophosphorylase in the *Aeromonas* genomes available. The *Aeromonas* glycogen synthase is able to react with UDP or ADP-glucose, which is not the case of *E. coli* glycogen synthase only reacting with ADP-glucose. The *Aeromonas* surface glucan has a role enhancing biofilm formation. Finally, for the first time to our knowledge, a clear preference on behalf of bacterial survival and pathogenesis is observed when choosing to produce one or other surface saccharide molecules to produce (lipopolysaccharide core or glucan).

## Introduction

Mechanisms of glycogen formation have been largely studied for *Escherichia coli* [1]. Glucose-1-phosphate is the early precursor for glycogen synthesis, and it is first converted to ADP-glucose (ADP-Glc) in a reversible reaction catalyzed by ADP-glucose pyrophosphorylase (EC 2.7.7.27). The glucosyl unit of ADP-Glc is transferred to the nonreducing end of an α-1,4-glucan chain by glycogen synthase (EC 2.4.1.21), and α-1,6 linkages are generated by a branching enzyme (EC 2.4.1.18). UDP-glucose (UDP-Glc) is considered to be the glucosyl donor for glycogen synthesis in mammalian cells. Either ADP-Glc or UDP-Glc can serve as glucosyl donors in eukaryotic microorganisms and Plants [2].

The biosynthesis of UDP-Glc in *E. coli* involves the following enzymatic reactions:

1. through GalU, Glucose-1 phosphate + UTP → UDP-Glc + PPi.
2. through Gal E, UDP-Glc ↔ UDP-Gal (UDP-galactose).
3. through GalT, Glucose-1 phosphate + UDP-Gal → Galactose-1 phosphate + UDP-Glc.

Reactions 2 and 3 need the bacterial growth in galactose as a single carbon source.

**Figure 1:**
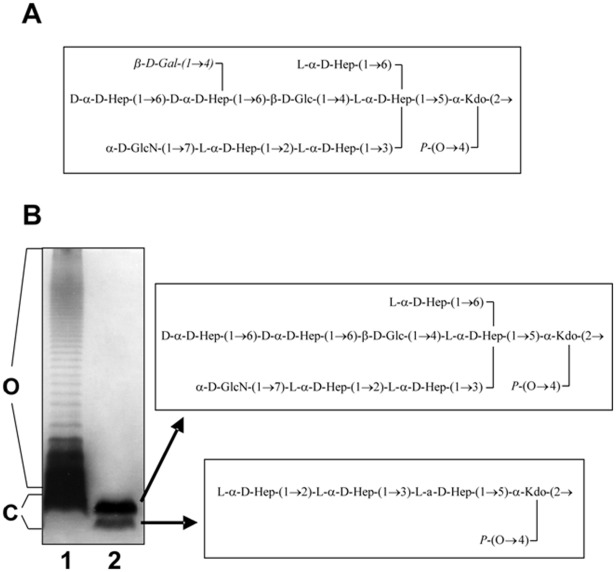
LPS of *Aeromonas hydrophila* AH-3. (A) Chemical structure of the LPS-core of *A. hydrophila* strain AH-3 [36]. The O34-antigen is linked to the Gal residue (shown in italics) of the LPS core [36]. (B) Silver-stained SDS-PAGE of purified LPS from *A. hydrophila* strain AH-3 (lane 1) and GalU mutant AH-2886 (lane 2) and structures of the two LPS-core bands of AH-2886 mutant [3]. All monosaccharides are in the pyranose form. Kdo, 3-deoxy-d-*manno*-oct-2-ulosonic acid; l-α-d-Hep, d-α-d-Hep, l-*glycero*- and d-*glycero*-α-d-*manno*-heptose; Glc, glucose; GlcN, glucosamine; Gal, galactose. C = LPS core, O = O34-antigen LPS.

*A. hydrophyla galU* mutants are unable to synthesize UDP-Glc from glucose-1-phosphate and UTP [3]. Besides its function as a substrate for glucosyltransferases resulting in glucosylated surface structures, UDP-Glc plays a well-established biochemical role as a glycosyl donor in the enzymatic biosynthesis of carbohydrates. The LPS from several *Aeromonas galU* mutants, either growing in rich or minimal media with glucose as a single carbon source, showed the absence of the O-antigen LPS and a LPS-core with higher electrophoretic mobility (two bands) as compared with the corresponding unique band of their respective wild type strains LPS-core [3]. Taking advantage of the complete determined LPS structure of strain AH-3 (serotype O34) [4], [5] ( Figure 1A), it appears that the two LPS bands from AH-3 *galU* mutant correspond to one (the major product represented by the slow-migrating LPS band on the gels) which is the complete LPS-core but devoid of Gal residue in the outer core where the O34-antigen LPS is attached; and the other (the minor product represented by the fast-migrating LPS band on the gels) with a deeply truncated LPS-core structure restricted to one Kdo and and three l-*glycero*-d-*manno*-heptose (ld-Hep) residues (see Figure 1B). The low amount of UDP-Glc in the mutant respect to the wild type explains its inability to complete the LPS-core structure. In a reduced number of the LPS molecules the mutant is unable to transfer the Glc residue to the inner-core, and the LPS-core is not further extended (Figure 1B). In the majority of the LPS-core molecules, the mutant is able to incorporate the Glc residue to the inner core and part of the outer core is added (major LPS-core product, see Figure 1B). However, even in this case the mutant is unable to complete the LPS-core because it can not incorporate the terminal Gal residue, probably because as mentioned before in the mutant the amount of UDP-Glc is too low [3]. The lack of Gal residue explains why the mutant is unable to express the O34-antigen LPS, because this residue links the O:34-antigen LPS [3]. Complementation with the single *A. hydrophila galU* gene completely restored all the LPS defects [3].

In this work we show the origin of the UDP-Glc obtained by the *A. hydrophila* strain AH-3 GalU mutant through the presence of a surface polysaccharide (glucan), which represents a new way to obtain UDP-Glc in *Aeromonas* strains. This surface *Aeromonas* glucan seems to play a role in biofilm formation.

## Results

### *Aeromonas Hydrophila* Strains Produce a Cell-surface Polysaccharide (Glucan)

**Figure 2:**
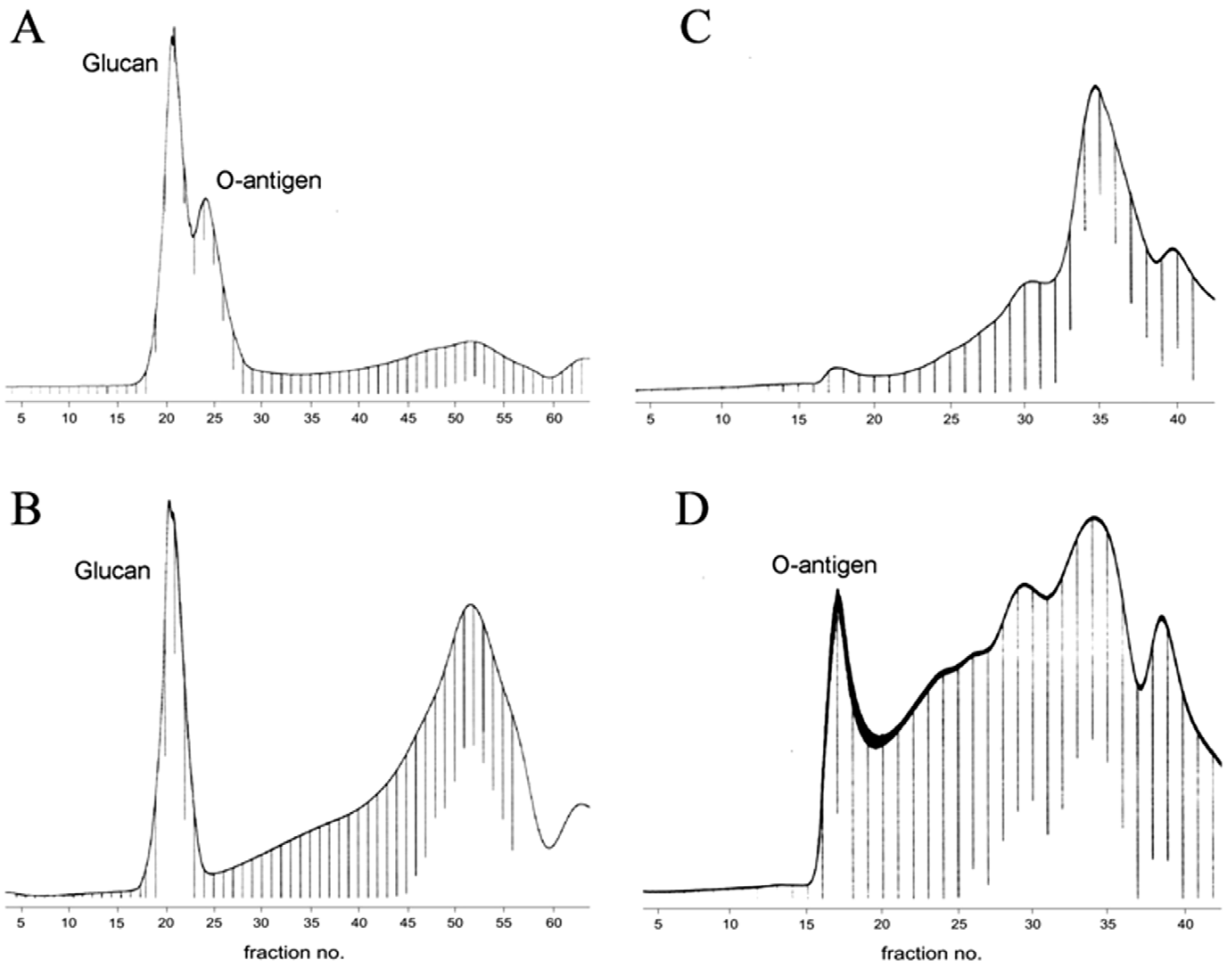
Sephadex G-50 gel permeation chromatography elution profiles of the water soluble (carbohydrate) material isolated by mild acid degradation of the LPS preparations from *A. hydrophyla* AH-3 (wild type) (A), AH-3Δ*rmlB* (B), AH-3Δ*wecP* (C) and AH-3Δ*glgA* (D) mutants.

A material containing cell-surface carbohydrates (LPS and a polysaccharide) was isolated from *A. hydrophila* strain AH-3 by the phenol-water extraction following digestion of cells with RNAse, DNAse and Proteinase K as described [4]. It was degraded with 1% acetic acid to cleave the lipid moiety of the LPS, and the resultant water-soluble (carbohydrate) material was fractionated by gel-permeation chromatography on Sephadex G-50 to yield glucan (eluted first) and then parts of the LPS: O-polysaccharide (O-antigen) and core oligosaccharide (Figure 2A).

**Figure 3:**
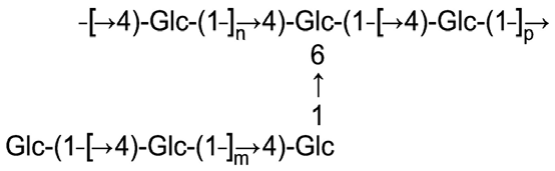
Structure representation of the branched glucan from *A. hydrophyla* AH-3 (wild type).

Sugar analysis of glucan showed the presence of D-glucose (Glc) only. The D configuration of glucose was established by GLC analysis of the acetylated (+)-2-octyl glycosides as described [5]. GLC was performed using an Agilent 7820A GC system and a temperature program from 160°C (1 min) to 290°C at 7°C min^–1^. Methylation analysis of glucan revealed terminal Glc, 4-substituted Glc and 4,6-disubstituted Glc in a ratio of 1∶5:1. Therefore, glucan is a branched polysaccharide. The ^1^H- and ^13^C-NMR spectra of glucan were assigned using two-dimensional ^1^H,^1^H homonuclear and ^1^H,^13^C heteronuclear correlation spectroscopy (COSY, TOCSY, HSQC, HSQC-TOCSY). They indicated that all glucose residues are α-linked; particularly, this followed from chemical shifts 4.95–5.35 ppm for H-1 and 99.9–101.3 ppm for C-1. The ^13^C-NMR spectrum showed also that ∼20% Glc residues are terminal (characteristic chemical shifts 71.0 ppm for C-4 and 62.4 ppm for C-6) and the rest are either 4-substituted (chemical shifts 79.2 ppm for C-4 and 62.3 ppm for C-6) or 4,6-disubstituted (chemical shifts 79.2 ppm for C-4 and 69.2 ppm for C-6). These data are in full agreement with methylation analysis data. A two-dimensional nuclear Overhauser effect NMR experiment showed that all terminal Glc residues are linked to position 4 of 4-substituted (or 4,6-disubstituted) Glc residues and none of them is linked to position 6 of 4,6-disubstituted Glc residues. 4-Substituted Glc residues are linked either to position 4 of 4-substituted (or 4,6-disubstituted) Glc residues or to position 6 of 4,6-disubstituted Glc residues. Based on these data together, the glucan structure can be represented as shown in Figure 3.

A RmlB mutant (AH-3ΔrmlB) devoid of O34-antigen LPS because the absence of 6-deoxy-L-talose [4] showed the presence of glucan in the soluble fraction after chromatography on Sephadex G-50 like the wild type strain AH-3, but not the O34-antigen LPS (Figure 2B). WecP is the enzyme that catalyzes the transfer of *N*- acetylgalactosamine to undecaprenyl phosphate to initiate the O-antigen lipopolysaccharide (LPS) biosynthesis [6]. No glucan or O34-antigen LPS [7] was obtained from the AH-3ΔwecP mutant cell surface (Figure 2C). WaaL is the enzyme that ligates the O-antigen LPS to the LPS-core [8]. The AH-3ΔwaaL mutant (AH-3Δ3.1 in [8]) showed no glucan or O34-antigen LPS.

When we studied different *A. hydrophila* wild type strains from different O-antigen serotypes, like for instance strain AH-1 (O11) or PPD134/91 (O18), we isolated a glucan polysaccharide identical to the one obtained from strain AH-3. In all these cases this glucan eluted before the LPS domains (O-polysaccharide and core oligosaccharide), independently of the O-antigen type (data not shown).

### The Glucan and the GalU Mutants

The AH-3 *galU* mutant (AH-2886) was devoid of the O34-antigen and showed two types of LPS structures corresponding to the two LPS bands on gels (Figure 1) [3]. When we tested for the presence of the glucan in the cell surface of AH-2886 mutant, we were unable to detect any polysaccharide by chromatography on Sephadex G-50 of the soluble fraction from the acid hydrolyzed phenol-water extract.

### The AH-3ΔglgA and AH-3ΔglgC Mutants: Glucan and LPS

The genome sequence of *A. hydrophila* ATCC7966^T^ [9] allow us to design primers to sequence the *glgA* and *C* region of *A. hydrophila* AH-3 (GenBank accession number JQ085284). The AH-3ΔglgA and AH-3ΔglgC mutants grown in a rich medium like LB were unable to produce glucan on the bacterial surface after the treatment with phenol-water and the chromatography on Sephadex G-50 of the soluble fraction, but were able to produce O34-antigen LPS (Figure 2D show the result for AH-3ΔglgA).

**Figure 4:**
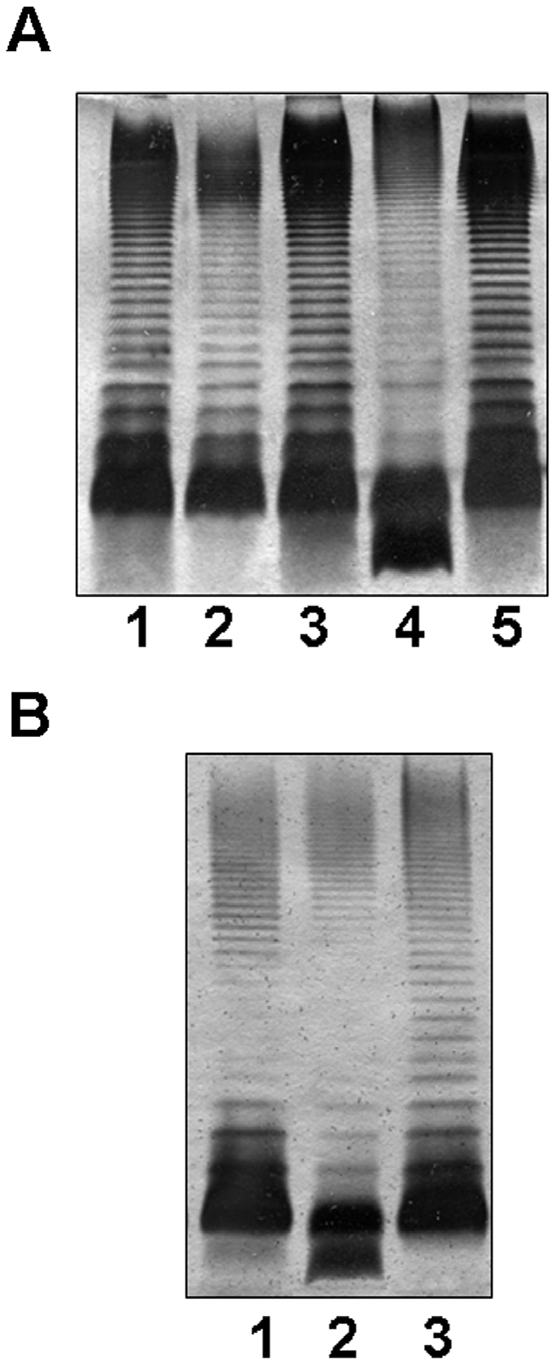
LPS of wild type strain and *glgA* and *C* mutants. (A) Silver-stained SDS-PAGE of the LPS from *A. hydrophyla* AH-3 (1), AH-3Δ*glgA* (2), and AH-3Δ*glgA* plus pBAD33-GlgA (3) when the strains were grown in LB. Lanes 4 and 5 show LPS from the same strains as 2 and 3, respectively, grown in minimal medium with glucose as a unique carbon source. (B) SDS-PAGE of the LPS from *A. hydrophyla* AH-3 (1), AH-3Δ*glgC* (2), and AH-3Δ*glgC* plus pBAD33-GlgC (3) strains grown in minimal medium with glucose as a unique carbon source.

The LPS profiles of both mutants, grown under the same conditions, analyzed on gels showed the presence of O34-antigen LPS with no significant differences with that of the wild type strain (Figure 4A lanes 2 and 3 show the results for AH-3ΔglgA mutant and wild type). However, when the mutant strains were grown on minimal medium with glucose as a unique carbon source, the LPS profiles of both mutants showed a reduced number of O34-antigen LPS molecules in comparison with the wild type strain and the appearance of two bands for the LPS-core (Figures 4A lane 4 and 4B lane 2). All these characteristics were rescued by the reintroduction of the *A. hydrophila* AH-3 *glgA* or *glgC* in a vector (pBAD33-GlgA or pBAD33-GlgC) (Figures 4A lane 5 and 4B lane 3), but not with the plasmid vector alone.

### The AH-3ΔgalU:glgA and AH-3ΔgalU:glgC Double Mutants

**Figure 5:**
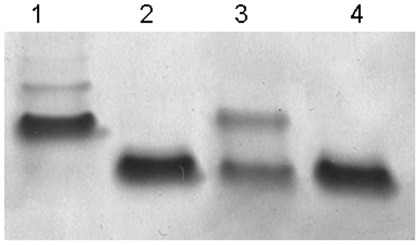
Silver-stained Tricine SDS-PAGE of the LPS from *A. hydrophyla* AH-3 (1), AH-3Δ*galU*:*glgA* double mutant (2), AH-3Δ*galU*:*glgA* plus pBAD33-GlgA from AH-3 (3), and AH-3Δ*galU*:*glgA* plus pBAD33-GlgA_Ec_ from *E. coli* (4).

**Figure 6:**
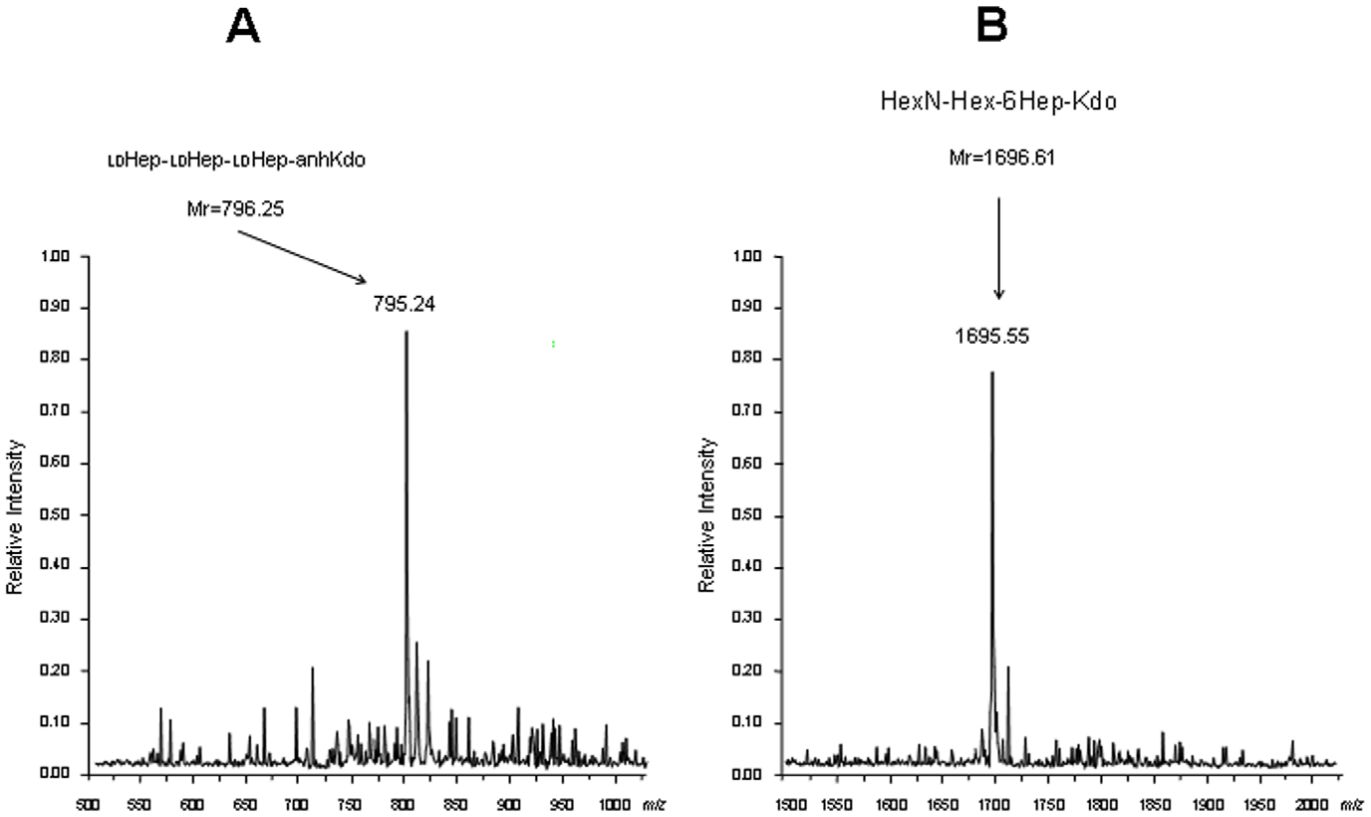
Negative ions ESI-MS of the core oligosaccharide LPS. (A) Core oligosaccharide obtained by acid release from the purified LPS of AH-3Δ*galU*:*glgA*. double mutant. (B) Core oligosaccharide obtained from the slow LPS migration band from AH-3Δ*galU*:*glgA* harbouring the AH-3 *glgA* (pBAD33-GlgA). anhKdo, an anhydro form of Kdo; M_r_, calculated molecular mass (Da).

From the AH-3 *galU* mutant (AH-2886) we obtained double mutants AH3ΔgalU:glgA and AH3ΔgalU:glgC as described in Materials and Methods. We were unable to detect the presence of glucan in the cell surface because they are *galU* mutants, but only a single LPS band (corresponding to the the one with faster migration in AH-3 *galU* mutant) could be detected on gels (Figure 5, lane 2, data for AH3ΔgalU:glgA). LPS was extracted and purified from strain AH-3ΔgalU:glgA, and the oligosaccharide fraction was obtained by mild acid hydrolysis (see Materials and Methods), and this fraction analyzed by Electrospray mass spectrum. A major ion peak for a Hep_3_Kdo compound with the molecular mass 795.24 Da was obtained (Figure 6A), in agreement with the LPS-core structure:

L-α-D-Hep-(1→2)-L-α-D-Hep-(1→3)-L-α-D-Hep-(1→5)-Kdo.

Similar results were obtained for AH3ΔgalU:glgC double mutant.

### Enzyme Activities

The results for UDP-Glc or ADP-Glc pyrophosphorylase and glycogen synthase enzyme activities, either using crude extracts or purified proteins from *A. hydrophila* AH-3 (wild type) or mutant strains, are summarized on Table 1. Briefly, wild type crude extracts showed UDP-Glc and ADP-Glc pyrophosphorylase activities but a reduced UDP-Glc pyrophosphorylase activity was obtained on crude extracts of the GalU mutant in comparison with the corresponding from the wild type strain. However, the UDP-Glc pyrophosphorylase enzyme activity on GalU mutant crude extracts was not abolished. The AH-3ΔglgC crude extracts showed a reduced UDP-Glc pyrophosphorylase enzyme activity, less than the ones obtained from the GalU mutant crude extracts, versus the wild type crude extracts. The crude extracts from the AH-3ΔglgA mutant showed no glycogen synthase activity while high activity was detected for purified AH-3GlgA. Purified AH-3 GlgC showed UDP-Glc but not ADP-Glc pyrophosphorylase enzyme activity. Furthermore, purified AH-3 GlgA glycogen synthase activity was largely inhibited by the presence of unlabelled UDP-Glc but not by unlabelled ADP-Glc.

**Table 1:** Enzymatic average values for *A. hydrophila* crude extracts and purified proteins.

Strain	UDP-Glc^a^ pyrophosphorylase	Sp act (nmol/min/mg of protein)	
		ADP-Glc^a^ pyrophosphorylase	Glycogen synthase
**AH-3 (wild type)**			
Crude extract	1.326+/–28	3.189+/–42	12+/–3
purified GlgA	ND	ND	52+/–14
purified GlgC	9,324+/–144	<1,0	ND
**AH-2286 (AH-3 ** ***galU*** ** mutant)**			
Crude extract	0.203+/–8	3.098+/–39	11+/–2
**AH-3**Δ**glgA**			
Crude extract	0.973+/–28	2.479+/–37	<0.2
**AH-3**Δ**glgC**			
Crude extract	0.935+/–21	2.971+/–40	11+/–3

*A. hydrophila* strains were grown under the conditions mentioned in Materials and Methods.

a Activity was measured in the pyrophosphorolysis direction.

Values are the average of three independent experiments +/– standard deviation.

ND, not determined.

### Complementation Studies Using AH-3ΔgalU:glgA Mutant

The enzyme activities results previously obtained prompted us to study the complementation of AH-3ΔgalU:glgA mutant with *glgA* from *A. hydrophila* AH-3 or *E. coli* through LPS studies. When plasmid vector carrying the AH-3 *glgA* (pBAD33-GlgA) was introduced into the AH-3ΔgalU:glgA mutant, two LPS-core bands without O34-antigen LPS were found (Figure 5, lane 3). The LPS migration profile in gel of this complemented mutant, was identical to that of GalU mutant, and the new band with slow migration on gel showed a major ion peak corresponding to a HexNHexHep_6_Kdo compound with a molecular mass 1696.61 (Figure 6B) in agreement with the LPS structure found in the single *galU* mutant [3] (see Figure 1B lane 2 upper band).

However, when the AH-3ΔgalU:glgA mutant was complemented with the same plasmid vector but carrying the *E. coli* DH5α *glgA* (pBAD33-GlgA_Ec_) no changes in its LPS profile was observed (Figure 5, lane 4). The only LPS-core band observed was the unique band for AH-3ΔgalU:glgA mutant according to its migration in the gel which correspond to the LPS chemical structure Hep_3_Kdo (Figure 6A).

### Adhesion to HEp-2 Cells and Biofilm Formation

No significant differences in adherence to HEp-2 cells were found between glucan producing or non-producing strains. Also, we compared the ability of the wild-type and mutant strains to form biofilms in microtiter plates (Table 2). The *A. hydrophila* wild-type strain, AH-3, exhibited a biofilm formation ability with an OD_570_ value of 1.32 ± 0.11. The mutant lacking only the O34-antigen LPS tested.

**Table 2:** Biofilm values of several *Aeromonas* strains using the method of Pratt and Kolter [35].

Strain	Value
AH-3 (wild type)	1.32+/–0.11
AH-3ΔrmlB (O^–^; glucan^+^)	0.65+/–0.07
AH-3ΔwecP (O^–^; glucan^–^)	0.47+/–0.05
AH-3ΔwecP + pBAD-WecP	1.29+/–0.13
AH-3ΔwaaL (O^–^; glucan^–^)	0.48+/–0.06
AH-3ΔwaaL + pBAD-WaaL	1.33+/–0.09
AH-3ΔglgA (O^+^; glucan^–^)	0.98+/–0.07
AH-3ΔglgA + pBAD33-GlgA	1.30+/–0.15
AH-3ΔglgA + pBAD33-GlgA_Ec_	0.96+/–0.10
AH-3ΔglgC(O^+^; glucan^–^)	0.99+/–0.08
AH-3ΔglgC + pBAD33-GlgC	1.30+/–0.14

(AH-3ΔrmlB) showed approximately a 55% reduction in their biofilm formation ability, while the mutants lacking both the O34-antigen LPS and surface glucan (AH-405ΔwecP or AH-3ΔwaaL) showed a reduction of 70%. Finally, the mutants with the O34-antigen LPS but unable to produce surface glucan (AH-3ΔglgA and AH-3ΔglgC) showed approximately a 25% reduction in their biofilm formation ability. The recovery of the O34-antigen LPS or surface glucan production in the mutants rescues the wild type strain value. This assay showed that surface glucan production has a role in biofilm formation, as well as O34-antigen LPS previously described by us as an adhesin [10].

AH-3ΔgalU:glgA double mutant (unable to produce O34-antigen LPS and the complete external LPS core, as well as glucan) was practically unable to form biofilm. It shows a drastic reduction (>85%) in their ability to form biofilm in comparison with the wild type strain using the same technique.

## Discussion

*A. hydrophyla* strain AH-3 GalU (UDP-Glc pyrophosphorylase) is responsible for synthesis of UDP-Glc from glucose-1-phosphate and UTP and *galU* mutants are unable to synthesize UDP-Glc by this reaction [3]. Chemical analysis of the LPS from these mutants showed the presence of glucose in a high percentage of the molecules isolated, which is not provided by GalU. This glucose residue is transferred by a β1,4 UDP-glucosyltransferase to the l-*glycero*-d-*manno*-heptoseII in the LPS inner-core. A second UDP-Glc pyrophosphorylase was found (GlgC), which together with the glycogen synthase GlgA is responsible for the biosynthesis of a branched α-glucan in strain AH-3. The UDP-Glc pyrophosphorylase GlgC was wrongly indicated as an ADP-Glc pyrophosphorylase in the complete *Aeromonas* genomes available [9], [11]. Therefore, in strain AH-3 the UDP-Glc can be synthesized via both GalU and GlgC. This situation is similar to the one described in some bacterial species, were there is a second UTP-glucose-1-phosphate uridyltransferase dedicated to the additional supply of UDP-Glc for specific purposes. Examples of this are the *Streptococcus hasC* supplying extra UDP-Glc for hyaluronic acid biosynthesis or the *Sphingomonas paucimobilis ugpG* performing the same role in gellan biosynthesis.

The *A. hydrophila* AH-3 α-glucan is a surface polysaccharide exported via the WecP which is also used by the O34-antigen LPS, and ligated to the surface through the O34-antigen polysaccharide ligase (WaaL). Nevertheless, it is an independent polysaccharide versus the O34-antigen LPS despite the common use of the export and ligation system, because we could find mutants devoid of either polysaccharide or both. The surface glucan is common to the mesophilic *Aeromonas* strains tested.

Bacteria, such as *Escherichia coli,* could show up to six distinct saccharide polymers simultaneously present within the glycocalyx. At present, the known components of the saccharide matrix include LPS O-antigens [12], enterobacterial common antigen (ECA) [13], capsular polysaccharides (K-antigen) [14], colanic acid (CA or M-antigen) [15], poly β-1,6-*N*-acetyl-D-glucosamine (PNAG) [16], and the β-1,4-glucan bacterial cellulose [17].

According to our results, the *Aeromonas* glucan production is via the glycogen synthase (GlgA) and the UDP-Glc pyrophosphorylase (GlgC). *A. hydrophila* AH-3 or ATCC7966^T^ (AHA_3804) GlgA showed a limited homology with *E. coli* GlgA, 38% identity and 52% similarity as the best result, and is the unique GlgA protein found in the *Aeromonas* complete genomes [9], [11]. *A. hydrophila* AH-3 or ATCC7966T (AHA_3803) GlgC showed also a rather limited homology with *E. coli* GlgC, 45% identity and 63% similarity as the best result, however two more *A. hydrophila* ATCC7966^T^ GlgC homologues named GlgC1 and GlgC2 were found in the *Aeromonas* ATCC7966^T^genome. These two *A. hydrophila* ATCC7966^T^ proteins GlgC1 (AHA_2482) and GlgC2 (AHA_2740) showed a higher homology (65% identity/82% similarity as the worse result) with *E. coli* GlgC than *A. hydrophila* GlgC.

The combination of the enzyme activities altogether with the LPS structures of the AH-3ΔgalU:glgA mutant and their complementation with *A. hydrophila* or *E. coli glgA*, supported the following considerations:

*A. hydrophila* AH-3GlgA is a glycogen synthase able to produce glucan through the addition of UDP-Glc and their analogue in *E. coli* is unable to do it. While *E. coli* GlgA only reacts with ADP-Glc in the glycogen synthesis, *A. hydrophila* GlgA reacts both with UDP-Glc (glucan synthesis) and probably with ADP-Glc (glycogen synthesis).
*A. hydrophila* AH-3 GlgC is an UDP-Glc pyrophosphorylase and should be renamed as this in the genome of strain ATCC7966^T^, because it is not the homologue for *E. coli* GlgC (ADP-Glc pyrophosphorylase). It seems that the *E. coli* GlgC homologues in *Aeromonas* strain ATCC7966^T^ are GlgC1 and GlgC2.
It is clear that *Aeromonas* is able to sense the drop of UDP-Glc generate by GalU mutation. When it happens, *Aeromonas* establish a preference for survival, abolish the production of surface glucan polysaccharide and produces a rather complete LPS-core by the incorporation of UDP-Glc molecules synthesized via GlgC (UDP-Glc pyrophosphorylase). To our knowledge it is the first report of a clear preference in choosing one or other surface saccharide molecule to produce on behalf of bacterial survival.

The results obtained indicated that surface glucan production may not have a significant role in cell adhesion but clearly has a role in biofilm formation. However, the O34-antigen LPS has a predominant role in both pathogenic characteristics: cell adhesion and biofilm formation. All of these surface polysaccharides characteristics also support the preference of the microorganism to produce LPS rather than surface glucan. Some *E. coli* exopolysaccharides (in particular CA and PNAG [18]) are integral components of biofilms, acting as the “cement,” which holds together the various protein, lipid, and polysaccharide components [19]. We suggested that a similar role may be played by *Aeromonas* surface α-glucan polysaccharide.

Several published reports indicate that the use of β-glucans enhances *Aeromonas* disease resistance in fish by potentiating innate immunity [20], [21]. The β-glucans used are from yeast representing a heterogeneous group of glucose polymers, consisting of a backbone of β-(1→3)-linked β-d-glucopyranosyl units with β-(1→6)-linked side chains of varying length and distribution. However, in no case the authors were able to show the scientific reason for this *Aeromonas* resistance. The fact that *Aeromonas* produces a surface α-glucan may explain these results, and also suggests that the use of α-glucans instead of β-glucans could be more helpful to enhance the fish resistance to *Aeromonas* disease.

## Materials and Methods

### Bacterial Strains, Plasmids and Growth Conditions

Bacterial strains and plasmids used in this study are shown in Table 3. *Aeromonas* were grown either in tryptic soy broth (TSB) or tryptic soy agar (TSA) and *E. coli* in Luria-Bertani (LB) Miller broth and LB Miller agar. Kanamycin (50 µg/ml), chloranphenicol (25 µg/ml) or ampicillin (100 µg/ml) were added to the different media when needed. When required the strains were cultured in Davis minimal medium [22] with glucose or galactose as the unique carbon source.

**Table 3:** Bacterial strains, and plasmids used.

Strain or plasmid	Relevant characteristics	Source or reference
***E. coli*** ** strains**		
DH5α	F^–^ *end A hsdR17* (r_K_^–^ m_K_^+^) *supE44 thi-1 recA1 gyr-A96* ϕ80*lacZ*M15	[37]
XL1-Blue	*recA1 endA1 gyrA96 thi-1 hsdR17 supE44 relA1 lac* (F^-^ *proAB lacI* ^q^ *Z*M15 Tn*10*)	Stratagene
S17-1	*hsdR pro recA*, RP4-2 in chromosome Km::Tn*7* (Tc::Mu)	[7]
BL21(λD3)	F^–^ *ompT hsdS_B_* (r_B_^–^ m_B_^–^) *gal dcm*(λD3)	Novagen
***A. hydrophila*** ** strains**		
AH-3	O34, Wild type	[7]
AH-405	AH-3, spontaneous Rif^R^	[7]
AH-2886	AH-3 *galU* mutant, Km^R^	[3]
AH-3ΔrmlB	AH-3 *rmlB* mutant in frame with pDM4	This study
AH-3ΔwecP	AH-3 *wecP* mutant in frame with pDM4	[6]
AH-3ΔwaaL	AH-3 *waaL* mutant in frame with pDM4	[8]
AH-3ΔglgA	AH-3 *glgA* mutant in frame with pDM4	This study
AH-3ΔglgC	AH-3 *glgC* mutant in frame with pDM4	This study
AH-3ΔgalU glgA	AH-2886 *glgA* mutant in frame with pDM4	This study
AH-3ΔgalU glgC	AH-2886 *glgC* mutant in frame with pDM4	This study
**Plasmids**		
pRK2073	Helper plasmid, Spc^R^	[7]
pBAD33	arabinose inducible expression vector, Cm^R^	ATCC
pBAD33-GlgA	pBAD33 with *A. hydrophila* AH-3 *glgA*	This study
pBAD33-GlgC	pBAD33 with *A. hydrophila* AH-3 *glgC*	This study
pBAD33-GlgA_Ec_	pBAD33 with *E. coli* DH5α *glgA*	This study
pDM4	*pir* dependent with *sacAB* genes, oriR6K, Cm^R^	[27]
pDM4Δ*rmlB*	pDM4 with *A. hydrophila rmlB* fragment, Cm^R^	This study
pDM4Δ*glgA*	pDM4 with *A. hydrophila glgA* fragment, Cm^R^	This study
pDM4Δ*glgC*	pDM4 with *A. hydrophila glgC* fragment, Cm^R^	This study
pET30 Xa/LIC	Vector for overexpressing His-tagged proteins	Novagen
pET30-glgA	pET30 overexpressing His6-GlgA strain AH-3	This study
pET30-glgC	pET30 overexpressing His6-GlgC strain AH-3	This study

### DNA Techniques

DNA manipulations were carried out essentially according to standard procedures [23]. DNA restriction endonucleases and *E. coli* DNA polymerase Klenow fragment were obtained from Promega. T4 DNA ligase and alkaline phosphatase were obtained from Invitrogen and GE Healthcare, respectively. PCR was performed using BioTaq DNA polymerase (Ecogen) in a Gene Amplifier PCR System 2400 Perkin Elmer Thermal Cycler.

### Nucleotide Sequencing and Computer Sequence Analysis

Double-stranded DNA sequencing was performed by using the dideoxy-chain termination method [24] with the Big Dye Terminator v3.1 cycle sequencing kit (Applied Biosystem). Oligonucleotides used for genomic DNA amplifications and DNA sequencing were purchased from Sigma-Aldrich. The DNA sequences were inspected in the GenBank and EMBL databases at the National Center for Biotechnology Information (NCBI) [25]. The Terminator search program in the GCG Wisconsin package was used to search for factor independent transcriptional terminators. ClustalW was used for multiple-sequence alignments [26].

### Construction of Defined Mutants

The chromosomal in-frame *rmlB*, *glgA*, and *glgC* deletion mutants, AH-3ΔrmlB, AH-3ΔglgA and AH-3glgC, respectively, were constructed by allelic exchange as described Milton et al. [27]. The primers used to obtain the mutants are listed in Table 4. Two asymmetric PCRs were carried out to obtain two DNA fragments (A-B and C-D) that were annealed at their overlapping region, and PCR amplified as a single DNA fragment using primers A and D. DNA fragments AB and CD of each gene were annealed at the overlapping regions provided by the primers B and C and amplified as a single fragment using primers A and D (Table 2). The fusion products were purified, *Bgl*II digested (the *Bgl*II site is present in primers A and D), ligated into *Bgl*II-digested and phosphatase-treated pDM4 vector, electroporated into *E. coli* MC1061 (*λpir*), and plated on chloramphenicol plates at 30°C to obtain pDM4Δ*rmlB*, pDM4Δ*glgA* and pDM4Δ*glgC* plasmids. Plasmids with mutated genes were transferred into *A. hydrophila* AH-405 rifampicin-resistant (Rif^r^) by triparental matings using the *E. coli* MC1061 *(λpir*) containing the insertion constructs and the mobilizing strain HB101/pRK2073. Transconjugants were selected on plates containing chloramphenicol and rifampicin. PCR analysis confirmed that the vector had integrated correctly into the chromosomal DNA. After sucrose treatment, transconjugants that were rifampicin resistant (Rif^R^) and chloramphenicol sensitive (Cm^S^) were chosen and confirmed by PCR.

**Table 4:** Primers used in the construction of chromosomal in-frame deletion mutants.

Primersa,b	Amplified Fragment
***rmlB***	
A: 5′- GAAGATCTCTTAGCACTCAACCCCTGT-3′	
B: 5′-*TGTTTAAGTTTAGTGGATGGG*ATCCCAGATGATATGACGAA3’	AB (595 bp)
C: 5′-*CCCATCCACTAAACTTAAACA*AAGACGGTGCAGTGGTATC-3′	
D: 5′- GAAGATCTACTGGTGATTTCCAACTCG-3′	CD (698 bp)
***glgA***	
A: 5′- GAAGATCTGCTCTATCGCGAGCTCAA-3′	
B: 5′- *TGTTTAAGTTTAGTGGATGGG*TTCCGAGGCAACAAACAG-3′	AB (638 bp)
C: 5′- *CCCATCCACTAAACTTAAACA*ATGAACACCCGCTTCAACT-3′	
D: 5′- GAAGATCTGGAACGCATTGCTCTGTTT-3′	CD (714 bp)
***glgC***	
A: 5′- GAAGATCTGGGTCATTGGATCTGTGCT-3′	
B: 5′- *TGTTTAAGTTTAGTGGATGGG*CTGAAGACGGGTACCTTCG-3′	AB (594 bp)
C: 5′- *CCCATCCACTAAACTTAAACA*GTGATCGGTGAAAATCTGGA-3′	
D: 5′- GAAGATCTGCATTTGAGCAGATTGACG-3′	CD (718 bp)

a Italic letters show overlapping regions.

b Underlined letters show *BglII* restriction site.

A double mutant *galU* and *glgA* (AH-3ΔgalU:glgA) was obtained using AH-3 *galU* mutant (AH-2886) and pDM4Δ*glgA* plasmid as mentioned above.

### Plasmid Constructions and Mutant Complementation Studies

For complementation studies, the *A. hydrophila* AH-3 genes *rmlB*, *glgA* and *C*, and *E. coli glgA* were PCR-amplified by using specific primer pairs and ligated to the plasmid pBAD33 (see the list of primers in Table 5). The plasmid constructions were transformed into *E. coli* LMG194 by electroporation, plated on chloramphenicol LB agar plates and incubated at 30°C. Plasmids with the amplified genes were independently transferred into the corresponding mutants by triparental mating using the mobilizing strain HB101/pRK2073. Transconjugants were selected on plates containing chloramphenicol and rifampicin and confirmed by PCR. Each gene was expressed from the arabinose-inducible and glucose-repressible promoter (p_BAD_) on pBAD33. Repression from the *araC* promoter was achieved by growth in medium containing 0.2% (w/v) D-glucose, and induction was obtained by adding l-arabinose to a final concentration of 0.2% (w/v). The cultures were grown for 18 h at 30°C in TSB medium supplemented with chloramphenicol and 0.2% glucose, diluted 1∶100 in fresh medium (without glucose) and grown until they reached *A*_600 nm_ of about 0.2. Then l-arabinose was added, and the cultures were grown for another 2 h. Repressed controls were maintained in glucose-containing medium.

**Table 5:** Primers used for mutant complementation using vector pBAD33.

Plasmid	Primer
**pBAD33-GlgA** a	
	A3glgAF1: 5′- TCCCCCGGGCTCGGGGATGTGAAGATTG -3′
	A3glgAR1: 5′- CCCAAGCTTGTTCAAAGCCGTGCTAAGG -3′
**pBAD33-GlgC** b	
	PRIMER FOR: 5′-GATATCTTCCGGGATCTGATAAGG-3′
	PRIMER REV: 5′- CCCAAGCTTAGATGCGATAGCCGATGT-3′
**pBAD33-GlgA_Ec_** c	
	w3310 glgA F1: 5′ TCCCCCGGGAAAACGCAGAGGAAGATGC-3′
	w3310 glgA R1: 5′- CCCAAGCTTCGTGGGCGATGAATATGTA-3′

a Primers contain *Sma*I(bold) and *Hind*III(underlined), the PCR amplified product (1668 bp) was ligated to *Sma*I- *Hind*III digested pBAD33.

b Primers contain *EcoR*V(bold) and *Hind*III(underlined), the PCR amplified product (1559 bp) was ligated to *Sma*I- *Hind*III digested pBAD33.

c Primers contain *Sma*I(bold) and *Hind*III(underlined), the PCR amplified product (1267 bp) was ligated to *Sma*I- *Hind*III digested pBAD33.

### LPS Isolation and Electrophoresis

LPS was extracted from dry cells grown in LB. The phenol/chloroform/light petroleum ether method [28] was used for strains producing rough LPS, while the phenol/water procedure [29] was used for the strains producing the O antigen domain (smooth LPS). For screening purposes LPS was obtained after proteinase K digestion of whole cells [30]. LPS samples were separated by SDS-PAGE or N-[2-hydroxy-1,1-bis(hydroxymethyl)ethyl]glycine (Tricine)-SDS-PAGE and visualized by silver staining as previously described [31].

### Large-scale Isolation and Mild Acid Degradation of LPS

Dry bacterial cells of each mutant in 25 mM Tris·HCl buffer containing 2 mM CaCl_2_ pH 7.63 (10 mL g^–1^) were treated at 37°C with RNAse, DNAse (24 h, 1 mg g^–1^ each), and then with Proteinase K (36 h, 1 mg g^–1^). The suspension was dialyzed, lyophilized, and the LPS extracted by the phenol-water procedure [29]. A portion of the LPS (∼50 mg) from each strain was heated with aqueous 2% HOAc (6 mL) at 100°C for 45 min. The precipitate was removed by centrifugation (13,000 *g*, 20 min) and the supernatant fractionated on a column (56 × 2.6 cm) of Sephadex G-50 Superfine in 0.05 M pyridinium acetate buffer pH 4.5 with monitoring using a differential refractometer (Knauer, Germany).

### Methylation Analysis

A sample of each oligosaccharide (0.5 mg) was dissolved in 1 mL dimethyl sulfoxide. An excess of powdered NaOH was added, the reaction glass flushed with dry N_2_, and sealed. After stirring at 20°C for 1 h, 0.5 mL of cold CH_3_I was added and the mixture stirred at 20°C for 1 h. Then water was added, the methylated product extracted with CHCl_3_, the extract washed with water, and evaporated with a stream of dry nitrogen. The methylated oligosaccharide was hydrolysed with 2 M CF_3_CO_2_H. (120°C, 2 h) and acid was removed with a stream of nitrogen. The methylated monosaccharides were conventionally reduced with NaBH_4_, acetylated with Ac_2_O in pyridine, and analysed by GLC on a Hewlett-Packard 5880 chromatograph and GLC-MS on a Hewlett-Packard HP 5989A instrument using a HP-5ms capillary column and a temperature gradient of 150°C (3 min) to 290°C at 7°C min^–1^.

### Mass Spectrometry

Electrospray MS was performed on a Micromass ZQ mass spectrometer (Waters) The sample (100 pmol) was deionized on Dowex H+ resin (Fluka) and dissolved in 2% triethylamined in 50% acetonitrile and injected in the ion source at a flow rate of 5 µl/min. The spectrum was acquired in the negative mode.

### Purification of *A. hydrophila* AH-3 His_6_-GlgA and GlgC

For *glgA* and *glgC* overexpression the pET-30 Xa/LIC vector (Novagen) and AccuPrime (Invitrogene, High-fidelity) polymerase were used. The *A. hydrophila* AH-3 *glgA* was amplified from genomic DNA using primers PET-A3glgA-for 5′- GGTATTGAGGGTC GCTTGGCTATTAACCCACTGA-3′and PET-A3glgA-rev 5′- AGAGGAGAGTTA GAGCCGTTCAAAGCCGTGCTAAG-3′, and for *A. hydrophila* AH-3 *glgC* primers PET-A3glgC-for 5′-GGTATTGAGGGTCGCATGGCAGGCA TATTCACCT-3′and PET-A3glgC-rev 5′- AGAGGAGAGTTAGAGCCCTTCAC AAGCCCCTCAACT-3′, and the PCR products independently ligated into pET-30 Xa/LIC (Novagen), and electroporated into *E. coli* BL21(*λ*DE3). The His_6_-GlgA and His_6_-GlgC proteins were overexpressed and cell lysates obtained as previously reported for other proteins [6]. The total membrane fraction was obtained by ultracentrifugation (200.000 × *g* 30 min at 10°C), the His_6_-GlgA and His_6_-GlgC proteins were solubilized and purified with a Ni^2+^-NTA agarose (Quiagen) as previously reported [6]. When needed the His_6_-GlgA and His_6_-GlgC proteins were concentrated using a Centriplus 10-ml YM-30 centrifugal filter device (Amicon Bioseparations), and typical protein preparations contained yields of 0.07–0.08 g/ml as determined by the Bio-Rad Bradford assay.

### Crude Extracts Production and Enzymatic Activity Measurements

Suspensions of bacteria (25% weight per volume), washed in 25 mM Tris-HCl buffer (pH 7.5) containing 1 mM MgCl_2_, and crude extracts obtained by passing the washed cells through a French pressure cell (1,120 kg/cm2) twice, and cellular debris removed by centrifugation (32,000 × *g* for 15 min at 4°C). Permeabilized cells were prepared by adding 25 ml of a toluene-ethanol mixture (1∶9 [vol/vol]) to 1 ml of cell suspension (approximately 3 mg of protein) as described previously [32]. Permeabilized cells were then pelleted by centrifugation (15,000 × *g* for 8 min at 4°C) and resuspended in 50 mM of Tris-HCl (pH 7.2). Protein concentrations of extracts were determined by using the Bio-Rad Bradford assay as directed by the manufacturer with bovine serum albumin as the standard.

NDP-glucose (ADP-glucose or UDP-glucose) pyrophosphorylase activities were determined spectrophotometrically by monitoring NADPH formation at 340 nm. Briefly, the reaction mixture (500 µl) contained 100 mM Tris-HCl (pH 7.2), 2.5 mM MgCl_2_, 2 mM fructose-1,6-bis phosphate, 10 mM sodium fluoride, 5 mM tetrasodium pyrophosphate, 1.5 U of phosphoglucomutase, 2 U of glucose-6-phosphate dehydrogenase, and 100 µl of crude extract (approximately 1 mg of protein) or purified AH-3 GlgC (50 µg ). The reaction was started by addition of 2 mM (final concentration) of ADP-glucose or UDP-glucose.

Glycogen synthase activity was determined by the incorporation of glucosyl units from UDP-[^3^H] glucose into glycogen [33]. The reaction mixture (600 µl) at 37°C included 50 mM Tris-HCl (pH 7.2), 3 mM MgCl_2_, 6 mM glucose-6-phosphate, 3.3 mM UDP-glucose, 10 µl of UDP-[^3^H]glucose (0.067 mCi/ml; 15.3 Ci/mmol), and permeabilized cells (approximately 1 mg of protein) or purified AH-3 GlgA (50 µg ). Aliquots (100 µl) were withdrawn at different times and immediately added to tubes containing 300 µl of 67.5% KOH to stop the reaction. Glycogen was isolated from this mixture by the ethanol precipitation. Dried glycogen was dissolved in 150 µl of H_2_O, and radioactivity was determined by liquid scintillation.

### Adherence Assay to HEp-2 Cell

The adherence assay was previously described [34]. Twenty HEp-2 cells/coverslips were randomly chosen, and the number of bacteria adhering/HEp-2 cell was recorded. Assays were carried out in triplicates.

### Biofilm Formation

Quantitative biofilm formation was performed in a microtiter plate as described previously [35], with minor modifications. Briefly, bacteria were grown on TSA and several colonies were gently resuspended in TSB (with or without the appropriated antibiotic); 100 µl aliquots were place in a microtiter plate (polystyrene) and incubated 48 h at 30°C without shaking. After the bacterial cultures were poured out, the plate was washed extensively with water, fixed with 2,5% glutaraldehyde, washed once with water and stained with 0,4% crystal violet solution. After solubilization of the crystal violet with ethanol-acetone (80/20, v/v) the absorbance was determined at 570 nm.

### Statistical Analysis

The data obtained in several assays were analysed by the *t-*test using Microsoft Excel software.
